# On the Treatment of the Various Forms of Acne and of Rosacea

**Published:** 1878-12

**Authors:** 


ON THE TREATMENT OF THE VARIOUS FORMS OF ACNE AND OF ROSACEA. By R. W. Taylor, M.D., (American Clinical Lectures, No. 34.) New York: G. P. Putnam's Sons. pp. 32.

This is a good, practical description of the symptoms and treatment of these very annoying and troublesome affections, by one of the most accurate and pains-taking dermatologists in this country. It will well repay perusal. The treatment recommended for acne is simple, easily carried out, and, as we can testify from our own experience, effective.

